# The circular RNA circ_0030018/miR-136/migration and invasion enhancer 1 (MIEN1) axis promotes the progression of polycystic ovary syndrome

**DOI:** 10.1080/21655979.2022.2041796

**Published:** 2022-02-19

**Authors:** Jing Xu, Qinghua Qu, Bao Liu, Liyuan Shen

**Affiliations:** a Department of General Gynecology, Chongqing Health Center for Women and Children, Chongqing China; b Department of Gynecological Endocrinology, Chongqing Health Center for Women and Children, Chongqing China

**Keywords:** PCOS, circ_0030018, miR-136, MIEN1, KGN cells

## Abstract

The abnormal expression of circular RNAs (circRNAs) is associated with the progression of polycystic ovary syndrome (PCOS), which commonly causes infertility in women. In this study, we identified the role of circ_0030018 in PCOS. Quantitative polymerase chain reaction (qPCR) was used to detect the expression levels of circ_0030018, microRNA (miR)-136, and migration and invasion enhancer 1 (MIEN1). Cell counting kit-8 and 5-ethynyl-2’-deoxyuridine assays were performed to analyze the proliferation of KGN cells. Apoptosis was analyzed using fluorescence-activated cell sorting. Transwell assays were performed to measure the migration and invasion abilities of cells. qPCR and Western blotting were used to measure the levels of E-cadherin, N-cadherin, Snail, and vimentin. The correlation of circ_0030018 or MIEN1 expression with miR-136 expression was confirmed via luciferase reporter and RNA pull-down assays. Results showed that circ_0030018 expression was upregulated in patients with PCOS and KGN cells. Knockdown of circ_0030018 suppressed the proliferation, migration, and invasion of cells, while promoting their apoptosis. circ_0030018 sponged miR-136, which targeted MIEN1. Moreover, downregulation of miR-136 abrogated the effects of circ_0030018 silencing, while the overexpression of MIEN1 reversed the miR-136-induced effect on KGN cells. In summary, loss of circ_0030018 delayed the progression of PCOS via the miR-136/MIEN1 axis.

## GRAPHICAL ABSTRACT

**Figure:**
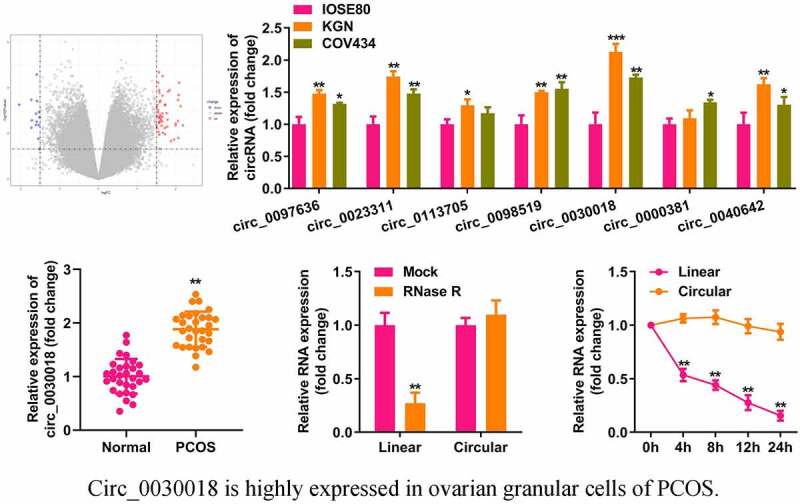

## Introduction

Polycystic ovary syndrome (PCOS), an endocrine disease common in women of childbearing age, has a prevalence of approximately 10%, affecting many families globally [1]. It is characterized by polycystic ovaries, hairiness, and anovulation. PCOS causes many other health problems, such as infertility, diabetes mellitus, obesity, cardiovascular diseases, and even malignancy [2,3]. At present, the etiology of PCOS is not fully understood, and genetic and environmental factors are considered the major pathogenic factors [4]. In addition, currently, there is no cure for PCOS. One way to alleviate PCOS is to control hyperandrogenism [5]. Thus, the molecular mechanisms underlying the pathogenesis of PCOS need to be elucidated. Abnormal growth and death of ovarian granular cells (GCs) are associated with PCOS progression [6]. Therefore, inhibiting the biological function of GCs may be an effective strategy for attenuating PCOS development.

Non-coding RNAs (ncRNAs) are dysregulated in patients with PCOS, especially in plasma, follicular fluid, and GCs. Differentially expressed ncRNAs are closely associated with steroid abnormalities, abnormal adipocytes, and GC function [7]. Circular RNAs (circRNAs) are highly conserved and stable ncRNAs. They commonly exert biological functions by serving as miRNA sponges or protein inhibitors [8,9]. Evidence has implicated that circRNAs regulate various physiological and pathological processes. CircRNAs have been implicated in diseases such as cardiovascular diseases, neurological diseases, immune diseases, and cancers [10,11]. According to microarray and RNA sequencing, numerous aberrantly expressed circRNAs are found in the cumulus cells and GCs of patients with PCOS and are involved in the development of PCOS [12,13]. Circ_0030018 (circ-POSTN), located in the chr13, has a 2656 bp spliced seq length. Recent research has revealed that circ_0030018 functions as a tumor promoter [^14–16^]. However, whether circ_0030018 plays a role in PCOS remains unknown.

CircRNAs commonly exert their roles by sponging microRNAs (miRNAs). MiRNAs are small non-coding RNAs that regulate target mRNA expression. Similar with circRNAs, miRNAs are commonly dysregulated in human diseases and regulate cellular processes such as proliferation, differentiation, and apoptosis [17]. MiR-136 is associated with numerous human diseases, such as malignancy, inflammation, gestational diabetes, neuropathic pain, and pre-eclampsia [^18–22^]. However, the role of miR-136 in PCOS needs further studied.

MIEN1, located on chromosome 17q21-22, is also known as C17orf37, C35, ORB3, RDX12, and XTP4. It is often amplified along the adjacent genes, ErBB2 and GRB7. MIEN1 is an overexpressed membrane-associated protein found in human cancers. It promotes tumor cell metastasis by regulating AKT activity [23]. A previous study has revealed that miR-136 targets MIEN1 to regulate metastasis in colon cancer [24]. However, the function of miR-136/MIEN1 in PCOS remains unclear.

In this study, we aimed to investigate the role of circ_0030018 in PCOS and the molecular mechanism. We hypothesized circ_0030018 is highly expressed in PCOS, and mediated the progression of PCOS by the miR-136/MIEN1 axis. The goal of this study provides a theoretical basis for circ_0030018 as a target for PCOS treatment.

## Materials and methods

### Microarray assay

Microarray GSE145296 data [12] were acquired from the Gene Expression Omnibus database (https://www.ncbi.nlm.nih.gov/geo/query/acc.cgi). The data were normalized using R language. Differentially expressed circRNAs were identified by |logFC| ≥ 1.5 and P value ≤ 0.05.

### Subjects

Ethical certification was obtained from the Ethics Committee of the Chongqing Health Center for Women and Children (code: 2,018,010; Chongqing, China). Written informed consent was obtained from all participants. Patients with PCOS (n = 30) and healthy subjects (n = 30) were enrolled in the study. The patients with PCOS were diagnosed based on the revised Rotterdam criteria [25]. The included patients with PCOS meets at least two of the following criteria: chronic oligo-ovulation or anovulation, androgen excess and polycystic ovaries. The patients with Cushing’s syndrome, congenital adrenal hyperplasia, androgen secreting tumors and endometriosis were excluded. Healthy individuals had normal menstrual cycles, ovulation, and ovarian morphology. None of the subjects had any other gynecological diseases or history of any drug allergies. The clinical characteristics of patients with PCOS and healthy controls are summarized in Table 1.

**Table 1.** Clinicopathologic characteristics of study subjects

Clinicopathologic characteristics	Normal(n = 30)	PCOS(n = 30)	Total(n = 60)	*p***-value**
Age (years)	33.5 ± 6.3	37.6 ± 10.0	35.6 ± 8.5	0.0623
BMI(kg/m^2^)	21.2 ± 1.1	21.7 ± 0.9	21.5 ± 1.0	0.0684
E2 (pg/ml)	44.1 ± 3.7	69.9 ± 9.9	57.0 ± 15.0	<0.0001
Testosterone (ng/ml)	0.3 ± 0.0	0.7 ± 0.1	0.5 ± 0.2	<0.0001
Progesterone (ng/ml)	0.6 ± 0.2	0.8 ± 0.1	0.7 ± 0.2	0.0004
Antral follicle count	11.3 ± 1.3	23.1 ± 2.5	17.2 ± 6.3	<0.0001
Oocytes obtained	9.0 ± 2.6	18.2 ± 6.3	13.6 ± 6.6	<0.0001
DASS scores	87.8 ± 13.3	80.3 ± 13.6	84.0 ± 13.8	0.0339
GHQ scores	45.7 ± 6.0	42.9 ± 8.4	44.3 ± 7.4	0.1333

BMI, body mass index; E2, estradiol; DASS, depression anxiety and stress scale; GHQ, general health questionnaire.

### Collection of ovarian GCs

All patients received gonadotropin-releasing hormone agonists and recombinant follicle-stimulating hormone. The patients were administered human chorionic gonadotropin after two or more follicles ≥ 18 mm and serum E2 levels ≥300 pg/mL. After 36 h, 2 mL follicular fluid was obtained from the dominant follicles via a vaginal puncture. Follicular fluid was centrifuged, and the cells were resuspended in hyaluronidase. GCs were collected after adding the lymphocyte separation liquid and stored at – 80°C.

### Cell culture

KGN, COV434, and IOSE80 cells were purchased from the Chinese Academy of Sciences (Shanghai, China). All cells were maintained in Dulbecco’s modified Eagle’s medium (Gibco, Grand Island, NY) containing 10% fetal bovine serum (Gibco) and 1% penicillin/streptomycin under a humidified atmosphere at 37°C with 5% carbon dioxide (CO_2_).

### Quantitative polymerase chain reaction (qPCR)

Total RNA was extracted using TRIzol reagent (Invitrogen, Carlsbad, CA, USA) and quantified using the optical density (OD)_260_ to OD_280_ ratio. Total RNA (1 μg) was reverse-transcribed to cDNA using the QuantiTect Reverse Transcription Kit (Qiagen, Duesseldorf, Germany). The AceQ qPCR SYBR Green Master Mix (Vazyme, Nanjing, China) was used to perform qPCR on an ABI7500 Real-Time PCR system (Applied Biosystems, Foster City, CA). The cycling conditions were shown as follows: 95°C for 5 min (predegeneration), 40 cycles of 95°C for 10s and 60°C for 30s (cycling reaction), followed by 95°C for 15s, 60°C for 1 min, and 95°C for 15s (melting curve). The normalization of circ_0030018 and mRNA levels was performed using glyceraldehyde‐3‐phosphate dehydrogenase (GAPDH), and the normalization of miR-136 levels was performed using U6. The expression (fold-change) was calculated using 2^−ΔΔCt^ method. The specific sequences of primers were listed in Table 2.

**Table 2.** The specific sequences of primers used in qPCR

Name	Sense (5’-3’)	Anti-sense (5’-3’)
Circ_0097636	CTGATGGCTTGAAAGAGGTGC	TCCAGCCACAAGACTGAGAC
Circ_0023311	TGGGAAGCTTGCTGTCATCAT	TAATCCACCTCGTACCGCTG
Circ_0113705	TGGTCCCTGCCCTCATGTAA	AGAGTGACCGTACGTCCCAA
Circ_0098519	GGAGCTTCACCAGAATGCAC	AATGAATGATGCACCAGCAGC
Circ_0030018	TGGACTTGGGAACAGGACTT	CCAATTTGTGTAAGCACACGGT
Circ_0000381	AAAGACAACACAAGTGCCCA	CCATGGTGTTCCCCTTCAATG
Circ_0040642	GACCGATCTTTCGGGAAGGC	CAGAGCAACTAAGCCGCCAT
E-cadherin	TACACTGCCCAGGAGCCAGA	TGGCACCAGTGTCCGGATTA
N-cadherin	GTGCCATTAGCCAAGGGAATTCAGC	GGAGGATACTCACCTTGTCCTTGCG
Snail	CTGCAGGACTCTAATCCAG	CGAGAGACTCCGGTTCCTA
Vimentin	GACAATGCGTCTCTGGCACGTCTT	TCCTCCGCCTCCTGCAGGTTCTT
MiR-136	ACTCCATTTGTTTTGATGATGGA	ATTCTAGAGGCCGAGGCGGCCGACATG
MIEN1	CAGTGCTGTGGAGCAGT	GACGGCTGTTGGTGATCTTT
GAPDH	CCACTCCTCCACCTTTGAC	ACCCTGTTGCTGTAGCCA
U6	GCTTCGGCAGCACATATACTAAAAT	CGCTTCACGAATTTGCGTGTCAT

MIEN1, Migration and invasion enhancer 1; GAPDH, glyceraldehyde-3-phosphate dehydrogenase.

### RNase R and actinomycin D treatment

Total RNA (2 μg) was exposed to 3 U/mg RNase R (Lucigen, Middleton, WI) at 37°C for 15 min. Total RNA was extracted without RNase R in the mock group. For actinomycin D treatment, the cells were treated with actinomycin D (Sigma-Aldrich, St. Louis, MO, USA) for 0, 4, 8, 12, and 24 h. Finally, qPCR was used to detect circ_0030018 and the corresponding linear mRNA levels.

### Nucleus cytoplasm separation assay

The localization of circ_0030018 was analyzed after nucleus and cytoplasm separation using nuclear/cytosolic fractionation kit (Cell Biolabs). About 5 х 10^6^ cells were collected and resuspended using 500 μL Cytosol Extraction Buffer. After incubating on ice for 10 min, cell lysis buffer was added into the cells. Then the cells were centrifugated at 800 хg for 10 min, and cytoplasm was in the supernatant. To extract nuclear protein, cells were resuspended using 100 μL Nuclear Extraction Buffer for 30 min on ice. After centrifugation at 14,000 хg for 30 min, nucleus was in the supernatant. Then we detected circ_0030018 in cytoplasm and nucleus. GAPDH was the internal control of cytoplasm, and U6 was the internal control of nucleus.

### Fluorescence in situ hybridization (FISH)

To determine the position of circ_0030018 in KGN cells, RNA-FISH assay was performed as described previously [26]. Circ_0030018 probe was labeled with FITC. The cells were fixed with 4% paraformaldehyde, and washed with PBS. Then the cells were permeated with Triton X-100. The probe was mixed with the hybridization buffer, and incubated at 37°C overnight. DAPI staining is used for nuclear localization. The images were taken under a fluorescence microscope (Olympus, Tokyo, Japan).

### Cell transfection

Lipofectamine 3000 (Invitrogen) was used for transfection, according to the manufacturer’s protocol. The siRNA (Si)-circ_0030018, si-negative control (nc), overexpressing (oe)-MIEN1 vector, and oe-nc were synthesized by GenePharma (Shanghai, China). miR-136 mimic, NC mimic, inhibitor, and NC inhibitor were purchased from Ribobio (Guangzhou, China). Cells were transfected into KGN cells for 48 h.

### Cell counting kit (CCK)-8 assay

Cell proliferation was assessed as previous reported [27]. KGN cells were seeded into 96-well plates, followed by incubation for 0, 24, 48, and 72 h. Next, the cells were incubated with CCK-8 (10 μL; KeyGEN, Nanjing, China) for 4 h. Absorbance was measured at 450 nm using a microplate reader (BioTek, Winooski, VT, USA).

### 5-ethynyl-2’-deoxyuridine (EdU) assay

kFluor488 Click-iT EdU Detection Kit (KeyGEN, Nanjing, China) was used for this assay. Briefly, KGN cells were immobilized in 4% paraformaldehyde for 30 min. Subsequently, the cells were incubated with glycine solution for 5 min. Then 0.5% Triton X-100 was utilized to penetrate the cells for 20 min after washing. The cells were then incubated with EdU or 4’,6-diamidino-2-phenylindole dihydrochloride for 30 min. Stained cells were observed under a fluorescence microscope.

### Fluorescence-activated cell sorting (FACS) analysis

Cell apoptosis was analyzed using an annexin V-PE/7-amino-actinomycin D (7-AAD) apoptosis detection kit (KeyGEN). Briefly, KGN cells were collected, washed twice with phosphate-buffered saline, and added to a binding buffer (50 μL) containing 7-AAD (5 μL). After 15 min, the cells were incubated with the binding buffer (450 μL) supplemented with annexin V-PE (1 μL) for 15 min. Apoptosis was examined using a flow cytometer (Beckman Coulter, Miami, FL, USA) within 1 h.

### Transwell assay

Transwell assay was performed as described previously [27]. Matrigel-dependent Transwell assay was performed to analyze the cellular invasion, whereas transwell without Matrigel was used to analyze cellular migration. Complete medium (0.6 mL) was added to the lower chamber as a chemoattractant. Cells were added to pre-coated or non-coated upper chambers (Corning, Corning, NY, USA). After incubation at 37°C for 48 h, we removed the non-migrated and non-invaded cells using a swab. Cells on the bottom surface were immobilized with 4% paraformaldehyde and subsequently stained with 0.1% crystal violet. Cells were imaged under a light microscope (Olympus).

### Western blotting

Proteins were prepared using the radioimmunoprecipitation assay buffer and quantified using a bicinchoninic acid protein assay kit (Pierce, Rockford, IL, USA). Lysed proteins were separated by 12% sodium dodecyl sulfate-polyacrylamide gel electrophoresis and transferred onto polyvinylidene fluoride membranes. After blocking with western blocking buffer for 1 h, the membranes were incubated overnight at 4°C with the anti-E-cadherin, anti-N-cadherin, anti-snail, anti-vimentin, and anti-GAPDH antibodies. GAPDH was used as the internal reference. The membranes were incubated with the secondary antibody at room temperature for 2 h. Protein bands were observed using an enhanced ECL kit (Pierce). Gray analysis was performed using Image J software.

### Luciferase reporter assay

A fragment containing the circ_0030018 or MIEN1 3′-untranslated region (UTR) was generated and inserted into the pGL3 vector (Promega, Madison, WI, USA) to recombine the wild-type (wt) reporter plasmid. Similarly, mutant (mut) circ_0030018 or MIEN1 3′-UTR sequences were also inserted into the pGL3 vector. Each wt or mut plasmid was co-transfected with the miR-136 mimic and NC using Lipofectamine 3000. After transfection for 48 h, firefly and Renilla luciferase activities were examined using a dual-luciferase reporter assay kit (Promega).

### RNA pull-down assay

miR-136 and NC mimics were labeled with biotin. KGN cells were transfected with biotin-nc and biotin-miR-136 for 48 h. Next, the cells were lysed, and the lysates were incubated with streptavidin magnetic beads (New England Biolabs, Beverly, MA) for 3 h. They were then washed with the lysis, low salt, and high salt buffers. circ_0030018 and MIEN1 expression levels were determined after total RNA extraction using qPCR.

### Statistical analysis

Data are shown as the mean ± standard deviation (SD). Comparisons among groups were analyzed by one-way analysis of variance (ANOVA), and differences between groups were analyzed by Student’s t-test using GraphPad Prism 7. Statistical significance was set at P < 0.05.

## Results

In this study, we explored the role of circ_0030018 in PCOS and the molecular mechanism. We analyzed KGN cell proliferation, apoptosis, migration, invasion, and metastasis. We found circ_0030018 was increased in PCOS, which depletion suppressed the progression of PCOS by the miR-136/MIEN1 axis. The study provided a novel insight for PCOS treatment.

### Circ_0030018 is highly expressed in ovarian GCs in PCOS

**Figure 1.**
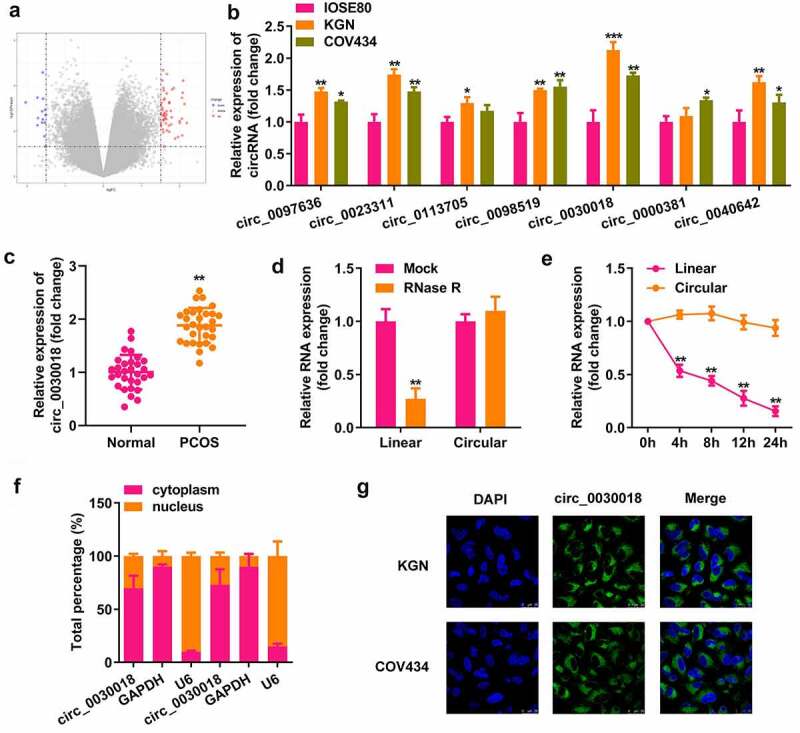
Circular RNA (circ_0030018) is highly expressed in the ovarian granular cells of patients with polycystic ovary syndrome (PCOS). (a) Differentially expressed circRNAs were predicted based on microarray GSE145296 and depicted using the volcano plot. (b) Expression levels of several circRNAs (circ_0097636, circ_0023311, circ_0113705, circ_0098519, circ_0030018, circ_0000381, and circ_0040642) were detected using quantitative polymerase chain reaction (qPCR) in IOSE80, KGN, and COV434 cells. (c) Expression levels of circ_0030018 were detected in patients with PCOS and healthy controls using qPCR. (d) Expression levels of circ_0030018 and corresponding linear mRNA were determined using qPCR after RNase R treatment. (e) The stability of circ_0030018 and corresponding linear mRNA was evaluated using qPCR after actinomycin D treatment. The localization of circ_0030018 was analyzed using (f) nucleus cytoplasm separation assay and (g) FISH. **P < 0.01. *P < 0.05.

Microarray analysis showed that multiple circRNAs were differentially expressed in the cumulus cells of patients with PCOS and healthy controls (Figure 1a). We found that the levels of seven abnormally expressed circRNAs were increased in KGN and COV434 cells compared with IOSE80 cells (Figure 1b). circ_0030018, which had the most significant elevation, was used in subsequent studies. circ_0030018 expression was significantly higher in patients with PCOS than in healthy subjects (P < 0.01; Figure 1c). circ_0030018 resisted RNase R degradation, which degraded the corresponding linear mRNA (P < 0.01; Figure 1d). After actinomycin D treatment, circ_0030018 was stable within 24 h, whereas the linear mRNA had a half-life of 4 h (P < 0.01; Figure 1e). As illustrated in figure 1f and G, circ_0030018 is mostly present in the cytoplasm of KGN and COV434 cells. Moreover, Table 1 lists the detailed clinical information of all subjects. PCOS is associated with estradiol, testosterone, progesterone, antral follicle count, oocytes, depression anxiety and stress, but it had little relation to age, BMI and other health condition.

### Loss of circ_0030018 suppresses the proliferation, migration, and invasion of cells, and induces their apoptosis

**Figure 2.**
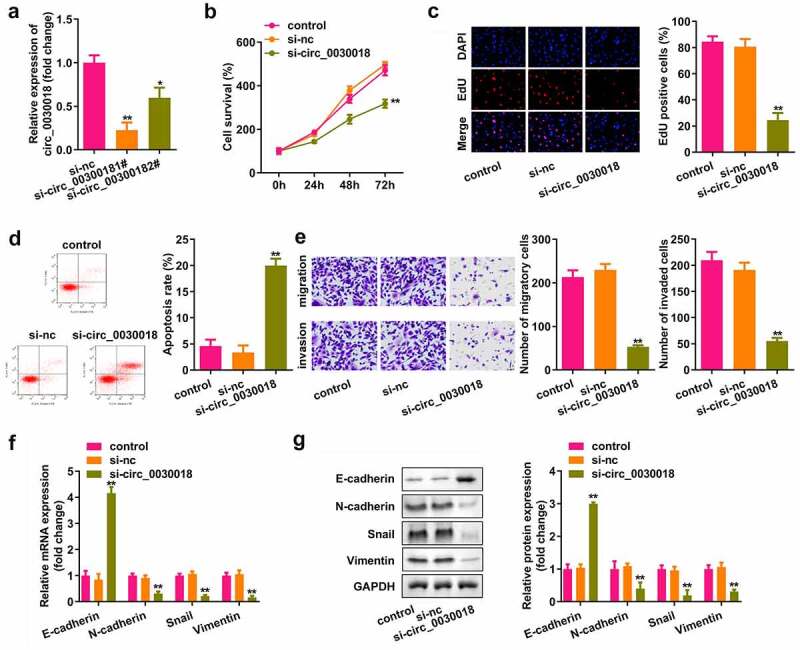
Loss of circ_0030018 suppresses the KGN cellular processes. (a) Expression levels of circ_0030018 were determined by qPCR after transfection. (b) Cell survival rate was tested using the cell counting kit-8 (CCK-8) assay. (c) Cellular proliferation was analyzed using 5-ethynyl-2’-deoxyuridine (EdU) assay. (d) Apoptotic cells were assessed using fluorescence-activated cell sorting (FACS) analysis. (e) Cellular migration and invasion were both analyzed using the Transwell assay. Expression levels of E-cadherin, N-cadherin, snail, and vimentin were detected using (f) qPCR as well as (g) Western blotting. Glyceraldehyde‐3‐phosphate dehydrogenase (GAPDH) was used as the internal reference. **P < 0.01.

Next, we assessed the role of circ_0030018 in KGN cells. Circ_0030018 was obviously downregulated after transfection with si-circ_0030018 1# (P < 0.01) and si-circ_0030018 2# (P < 0.05; Figure 2a). The cells transfected with si-circ_0030018 1# were used in this study. Knockdown of circ_0030018 significantly suppressed cell proliferation, facilitated apoptosis, and inhibited migration and invasion (all P < 0.01; Figure 2(b-E)). Moreover, knockdown of circ_0030018 significantly increased the expression of E-cadherin, while downregulating the levels of N-cadherin, snail, and vimentin (all P < 0.01; Figure 2(f-G)).

### Circ_0030018 targets miR-136

**Figure 3.**
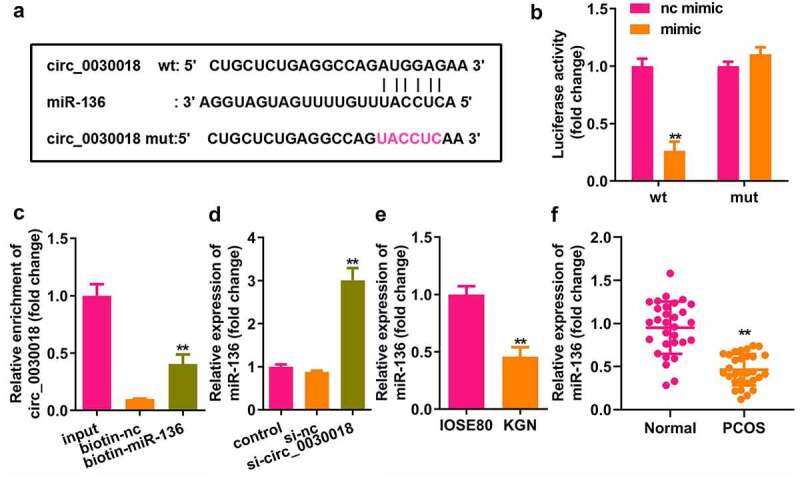
Circ_0030018 targets microRNA (miR)-136. (a) Bioinformatic analysis showed the binding sites between circ_0030018 and miR-136. (b) Luciferase activity of cells was examined after co-transfection with plasmids and mimic or the negative control (NC). (c) circ_0030018 was tested after the pull-down assay using biotin-miR-136 or biotin-nc. (d) miR-136 was examined using qPCR after the silencing of circ_0030018. (e) miR-136 expression levels were determined in IOSE80 and KGN cells by qPCR. (f) miR-136 expression levels were determined in patients with PCOS and healthy individuals by qPCR. **P < 0.01.

Bioinformatic analysis showed that circ_0030018 could bind to miR-136 (Figure 3a). MiR-136 mimic rather than nc mimic markedly decreased luciferase activity after co-transfection with wt-circ_0030018, whereas miR-136 co-transfection with mut-circ_0030018 did not affect luciferase activity (P < 0.01; Figure 3b). circ_0030018 was clearly captured by biotin-miR-136 compared to biotin-nc (P < 0.01; Figure 3c). Silencing circ_0030018 induced a significant elevation in miR-136 expression (P < 0.01; Figure 3d). The levels of circ_0030018 were significantly reduced in KGN cells and patients with PCOS (P < 0.01; Figure 3(e,F)).

### Depletion of circ_0030018 suppresses the progression of PCOS by sponging miR-136

**Figure 4.**
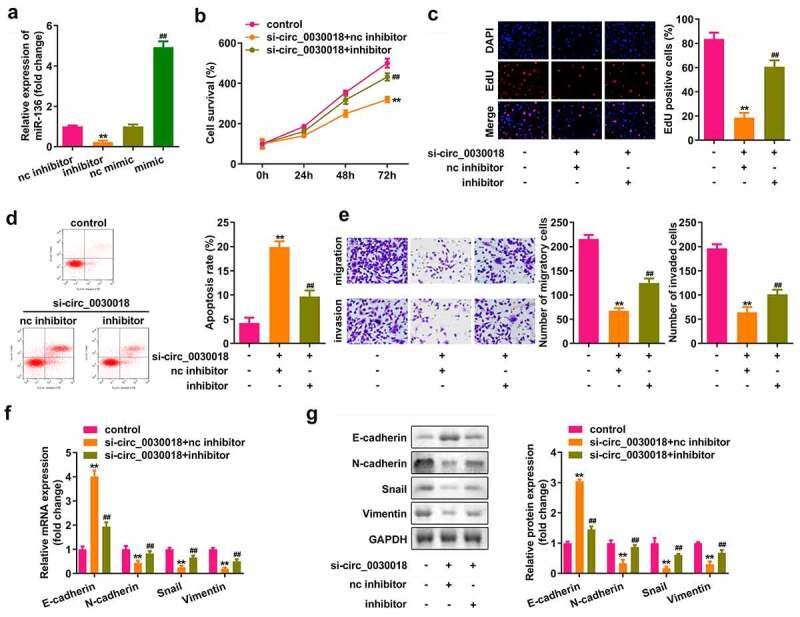
Depletion of circ_0030018 suppresses the progression of PCOS by sponging miR-136. (a) miR-136 expression levels were measured by qPCR post-transfection. (b) Cell survival was evaluated by the CCK-8 assay. (c) EdU assay was conducted to evaluate cellular proliferation. (d) Cell apoptosis was assessed using FACS analysis. I Transwell assay was used to evaluate the migration and invasion of cells. Expression levels of E-cadherin, N-cadherin, snail, and vimentin were determined by (f) qPCR and (g) their protein levels were determined by Western blotting. **P < 0.01. ##P < 0.01.

After transfection, miR-136 expression was significantly decreased in the inhibitor group, while miR-136 was significantly upregulated in the mimic group (P < 0.01; Figure 4a). Knockdown of circ_0030018 significantly suppressed cell proliferation, facilitated apoptosis, and inhibited migration and invasion, while the miR-136 inhibitor markedly abrogated the effects induced by circ_0030018 loss (all P < 0.01; Figure 4(b-E)). E-cadherin expression was markedly elevated, but N-cadherin, Snail, and vimentin expression was markedly decreased by circ_0030018 loss, which was reversed by inhibiting miR-136 (all P < 0.01; Figure 4(f,G)).

### miR-136 targets MIEN1

**Figure 5.**
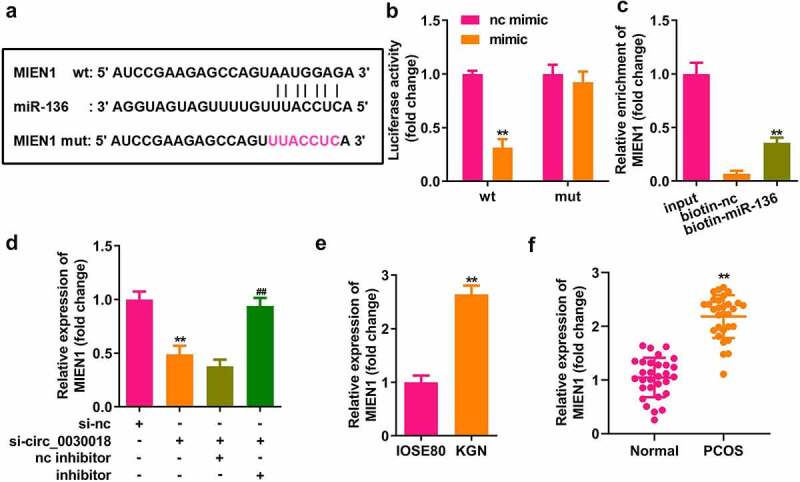
miR-136 targets the migration and invasion enhancer 1 (MIEN1). (a) The sequences of MIEN1 binding to miR-136 were predicted by bioinformatics analysis. (b) Luciferase activity was detected after co-transfection with the wild-type (wt) or mutant (mut) plasmids and mimic or NC, (c) MIEN1 levels were tested after pull-down assay with biotin-miR-136 or biotin-nc. (d) MIEN1 expression levels were determined using qPCR after silencing circ_0030018 and inhibiting miR-136. (e) MIEN1 expression levels were determined in IOSE80 and KGN cells. (f) MIEN1 expression levels were determined in patients with PCOS and healthy individuals. **P < 0.01.

Bioinformatics analysis indicated that MIEN1 has binding sites for miR-136 (Figure 5a). Compared with the NC mimic, miR-136 co-transfected with the wt plasmid markedly reduced luciferase activity. However, nc and miR-136 did not affect luciferase activity when co-transfected with mut plasmid (P < 0.01; Figure 5b). MIEN1 was pulled down by biotin-miR-136 (P < 0.01; Figure 5c). MIEN1 expression was markedly reduced by silencing circ_0030018 and was rescued by inhibiting miR-136 (both P < 0.01; Figure 5d). MIEN1 levels were significantly higher in KGN cells than in IOSE80 cells (P < 0.01; Figure 5e). Similarly, MIEN1 expression was obviously increased in patients with PCOS compared to that in healthy controls (P < 0.01; figure 5f).

### miR-136 attenuates the progression of PCOS by targeting MIEN1

**Figure 6.**
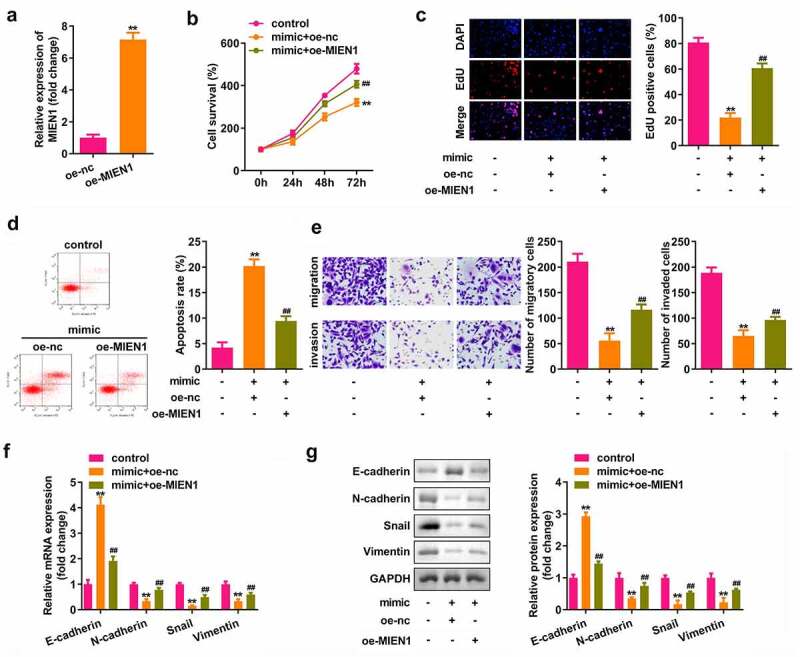
miR-136 attenuates the progression of PCOS by targeting MIEN1. (a) MIEN1 levels were determined using qPCR after transfection with the MIEN1 overexpression vector. (b) CCK-8 assay was performed to evaluate cell survival. (c) EdU assay was carried out to evaluate cellular proliferation. (d) Cell apoptosis was assessed using FACS analysis. (e) Transwell assay was performed to analyze the migration and invasion of cells. mRNA expression levels of E-cadherin, N-cadherin, snail, and vimentin were determined by (f) qPCR and (g) their protein levels were determined by Western blotting. **P < 0.01. ##P < 0.01.

As shown in Figure 6a, MIEN1 expression was upregulated after transfection with the MIEN1 overexpression vector (P < 0.01). Cell growth, migration, and invasion were significantly suppressed, but apoptosis was induced by miR-136, which was significantly rescued by MIEN1 (all P < 0.01; Figure 6(b-E)). Overexpression of miR-136 upregulated E-cadherin, but downregulated N-cadherin, Snail, and vimentin, while overexpression of MIEN1 abolished the miR-136-induced effect (all P < 0.01; Figure 6(f,G)).

## Discussion

Here, we clarified the role of circ_0030018 in PCOS and its underlying molecular mechanism. Circ_0030018 was elevated in PCOS and KGN cells, and its silencing impeded the biological progression of KGN cells. Moreover, circ_0030018 functions via the miR-136/MIEN1 axis.

Accumulating evidence has shown that circRNAs regulate PCOS occurrence and development. Recently, more and more circRNAs have been identified to be abnormally expressed in PCOS, and closely related to inflammation, apoptosis, and steroidogenesis [28,29]. The effects of circRNAs on GC have also been identified. For example, circPSMC3 suppresses KGN cell proliferation and facilitates apoptosis [30]. Interference with circASPH impedes cell growth and induces apoptosis [31]. Circ_0023942 inhibits the growth of GCs, thus alleviating the development of PCOS [32]. However, the role of circ_0040018 in PCOS remains unclear. Previous studies have shown that circ_0030018 promotes the progression of various cancers, including glioma and esophageal carcinoma. Silencing circ_0030018 suppresses tumor cell growth, metastasis, and apoptosis [^14–16^]. Herein, we found that circ_0030018 was highly expressed in patients with PCOS and in KGN cells, suggesting that it participates in PCOS development. Furthermore, knockdown of circ_0030018 regulated biological behaviors, suggesting that depletion of circ_0030018 alleviated PCOS progression.

CircRNAs commonly exert their roles by sponging miRNAs. We confirmed that miR-136 is a target of circ_0030018. MiR-136 is involved in numerous diseases [^18–22^]. Most research on miR-136 has focused on its role in cancers, especially its effect on biological functions. For instance, upregulated miR-136 expression decreases the proliferation and metastasis of osteosarcoma cells [18]. MiR-136 inhibits liver cancer cell metastasis [33]. MiR-136-5p, modulated by circ_RANBP9, decelerates the progression of PCOS [34]. In the present study, miR-136 was expressed at lower levels in patients with PCOS. Inhibition of miR-136 reversed the effect on the biological behavior induced by circ_0030018 loss, similar to previous studies [34,35]. These data indicated that silencing circ_0030018 attenuated PCOS progression by sponging miR-136.

MIEN1 has a thioredoxin-like fold and regulates AKT activity through its redox-active motif [36]. It is overexpressed in cancers and promotes cancer development. MIEN1 mainly exists in the cytoplasm and increases cell migration by inducing the formation of filamentous pseudopodia in the leading edge of migrating cells [23]. However, the role of MIEN1 in PCOS remains unclear. MIEN1 expression is increased in PCOS and it acts as a miR-136 target, which is consistent with the results of a previous study [18,24]. MIEN1 overexpression reversed the effects of miR-136. These findings suggest that miR-136 targets MIEN1 to promote PCOS development. Taken together, the loss of circ_0030018 attenuated PCOS progression via the miR-136/MIEN1 axis.

However, there are still limitations in this study. First, the number of clinical samples were small. Thus, it is hard to evaluate its clinical effect. Additionally, this study only focused on the role of circ_0030018 at the cellular level. We will study its role *in vivo* in our future work.

## Conclusion

In summary, depletion of circ_0030018 attenuated PCOS progression by modulating the miR-136/MIEN1 axis. These findings suggest that circ_0030018 has potential to act as a therapeutic target for PCOS.

## Data Availability

The datasets used and analyzed during the current study are available from the corresponding author on reasonable request.

## Research highlights

1. Circ_0030018 is highly expressed in ovarian granular cells of patients with PCOS.
2. Loss of circ_0030018 suppresses KGN cellular processes.
3. Circ_0030018 sponges miR-136 to target MIEN1.
4. Silencing of circ_0030018 inhibits cellular processes via the miR-136/MIEN1 axis.
